# Analysis of factors related to death of severe acute pancreatitis

**DOI:** 10.1186/1471-227X-12-S1-A8

**Published:** 2012-12-18

**Authors:** Chen Ji-jun, Li Chao, Wang Yan-jun, Zhao Bing-gang, Han Qiang, Yin Wen

**Affiliations:** 1 Department of Emergency Xijing Hospital, the Fourth Military Medical University, Changle Xilu Road No.127, Xi'an 710032, China

## Background

Severe acute pancreatitis (SAP) is defined as pancreatitis in the context of acute organ dysfunction, the mortality rate of which is still high. The present study was undertaken to investigate the risk factors of the mortality of SAP.

## Methods

Data of 312 patients of SAP admitted to our hospital from January 2010 to May 2012 were reviewed.All patients were divided into 2 groups, the death group and the survival group. The factors related to death of SAP were analyzed with Logistic regression analysis.

## Results

Sixty-seven of 312 patients (24.48%) died. There were significant differences between two groups in age, body mass index, length of stay hospital, APACHE II score, multiple organ dysfunction syndrome (MODS) and abdominal compartment syndrome, all *P*<0.05.

## Conclusions

MODS, especially respiratory dysfunction and renal dysfunction, is the main factors relating to mortality of SAP, whereas ACS, Balthazar CT class, infection, and age may impact death. Therefore early management of acute pancreatitis should be focused on the prevention and management of respiratory dysfunction and renal dysfunction.

